# A Comparison of the Adoption of Genomic Selection Across Different Breeding Institutions

**DOI:** 10.3389/fpls.2021.728567

**Published:** 2021-11-19

**Authors:** Mahmood Gholami, Valentin Wimmer, Carolina Sansaloni, Cesar Petroli, Sarah J. Hearne, Giovanny Covarrubias-Pazaran, Stefan Rensing, Johannes Heise, Paulino Pérez-Rodríguez, Susanne Dreisigacker, José Crossa, Johannes W. R. Martini

**Affiliations:** ^1^KWS SAAT SE & Co. KGaA, Einbeck, Germany; ^2^Genetic Resources Program, International Maize and Wheat Improvement Center, Texcoco, Mexico; ^3^Excellence in Breeding Platform, Consultative Group for International Agricultural Research, Texcoco, Mexico; ^4^IT Solutions for Animal Production (vit - Vereinigte Informationssysteme Tierhaltung w.V.), Verden, Germany; ^5^Department of Statistics, Colegio de Postgraduados, Montecillos, Mexico; ^6^Global Wheat Program, International Maize and Wheat Improvement Center, Texcoco, Mexico

**Keywords:** genomic selection, breeding, plant breeding, animal breeding, dairy breeding, selection gain

## Introduction

Within the last 20 years, after the landmark paper by Meuwissen et al. (2001), genomic selection (GS) has been widely incorporated in plant and animal breeding (Crossa et al., 2017; Hickey et al., 2017). However, adoption happened at different speeds and with distinct focus.

Here, we give a short description of the history and the current state of GS implementation in German dairy cattle breeding (as an example in animal breeding), at the private plant breeding company KWS SAAT SE & Co. KGaA, and at the public breeding programs of the International Maize and Wheat Improvement Center (CIMMYT) and the Consultative Group for International Agricultural Research (CGIAR) in general. We close by highlighting some differences in organizational structure and objectives of the considered breeding institutions, and comment on how these differences may have influenced the adoption of GS.

## Genomic Selection in Dairy Cattle Breeding

Dairy cattle breeding provided good conditions for the introduction of GS. Selection decisions had been based for decades purely on additive genetic effects reflected in a sire's breeding value, and the use of pedigree-based estimated breeding values (PEBVs) had already been common practice. However, reliabilities of early estimated breeding values from information on parents only were low. Therefore, a testing scheme was used, in which bulls were mated to a more or less representative sample of cows in a first step. The resulting daughters were then raised until their performance could be measured, thus improving the reliabilities of their sires' breeding values. Only then, the best test bulls were selected and used broadly. This costly waiting period led to a generation interval of more than five years. Using genomically estimated breeding values (GEBVs) of young bulls, which are more reliable than PEBVs, permitted to reduce this waiting period, and thus to increase selection gain per time. Although the accuracy of the breeding value of a bull which has been extensively progeny tested over years is of higher accuracy than a young bull's GEBV, the costs in terms of waiting time do not pay off for the breeding program, when comparing a more accurate late selection to a less accurate early selection based on the GEBV instead of the PEBV.

**Figure 1:**
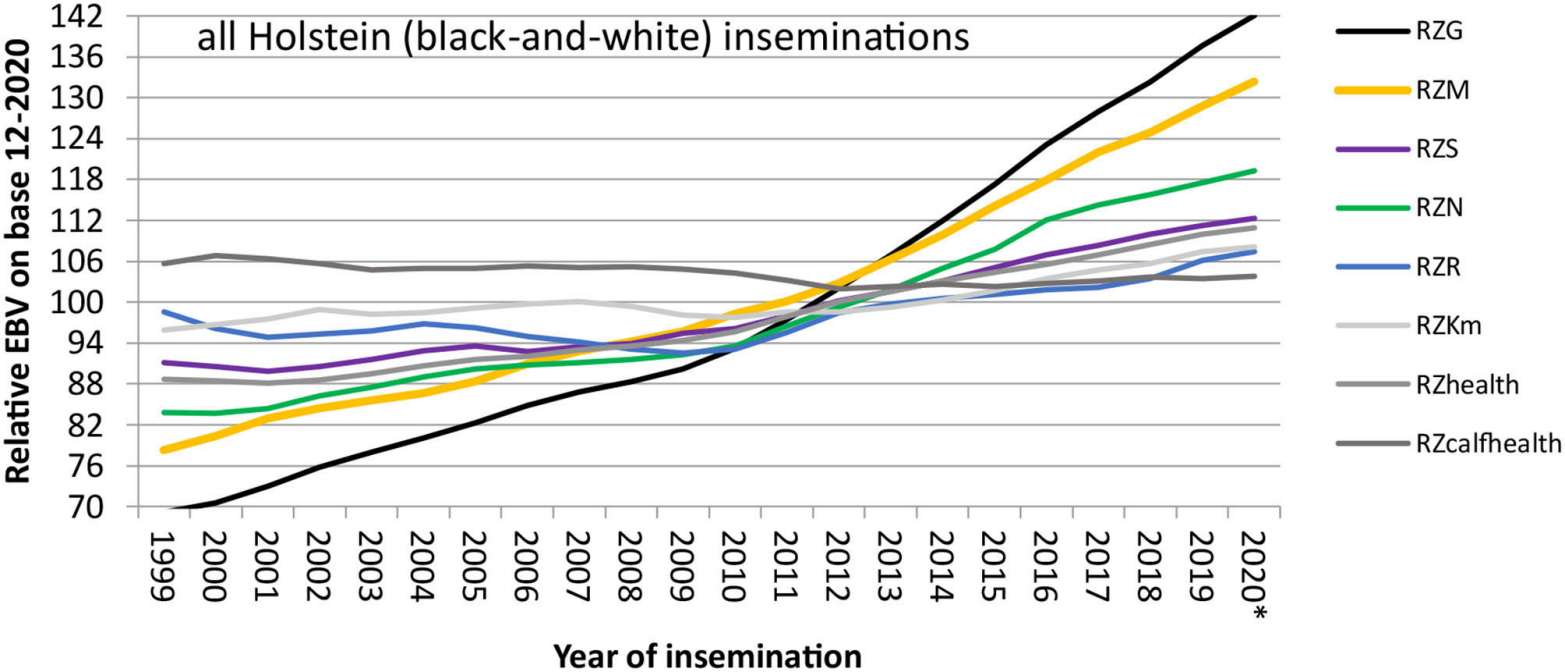
Breeding progress in important traits in German Holsteins, measured as yearly mean EBVs of bulls, weighted by the number of inseminations with their semen. The label “RZ” denotes that all breeding values are standardized to a genetic standard deviation of 12, and a mean of 100 in the female base population (year of birth 2014–2016), all breeding values are expressed such that more positive values are more desirable from the breeder's perspective. RZG, total merit index; RZM, milk production index; RZS, somatic cell score; RZN, longevity; RZR, fertility index; RZKm, index of maternal calving traits; RZhealth, health trait index; RZcalfhealth, calf survival. *Year 2020: incomplete data. Slightly modified from IT Solutions for Animal Production (vit - IT Solutions for Animal Production, 2021).

With this setup, genomic breeding values for Holsteins and Jerseys were first published in the USA in 2009 (Wiggans et al., 2017), about a decade after the release of the first commercial SNP chip (Wang et al., 1998). In Europe, four breeding organizations (UNCEIA: France; VikingGenetics: Denmark, Finland, and Sweden; DHV-vit: Germany; CRV: The Netherlands, Flanders) joined forces and put a reference population together with 4,000 bulls each (Lund et al., 2011). After 1.5 years of development, from August 2010 onwards, genomic breeding values, based on the joint reference population, were published in these European countries. This rapid evolution was only possible due to a long-established international data infrastructure with Multiple Across Country Evaluations (MACE) being in place since the 1990s at the international evaluation center Interbull. MACE allows the expression and use of estimated breeding values on the scale of each participating country (Schaeffer, 1994). Since 2010, breeding progress has more than doubled for all traits in German Holsteins as seen from Figure 1, mostly due to the sharply decreased generation interval for bulls.

The initial 50k Illumina SNP set is still the reference SNP set for genomic evaluations at vit in Germany, although dozens of different SNP chips have been integrated since then, especially many low density chips. With dropping genotyping costs and low density 10k SNP chips, female animals came also into the focus. In 2019, cows were integrated in the German reference population. As of the routine genetic evaluation in April 2021, there were 43,699 bulls and 249,363 cows in the reference population for milk traits. Current efforts aim at implementing Single Step methodology (Aguilar et al., 2010) in the genetic evaluation systems of most countries, which is a computationally demanding task with big populations, requiring specialized algorithms (e.g., Liu et al., 2014).

## Genomic Selection at KWS

Around 2008, KWS started own research activities in the field of GS and participated in several large collaborations (e.g., Albrecht et al., 2011; Hofheinz et al., 2012). Only a few years later, GS became an established part of the breeder's toolbox for all KWS field crops.

The reason for this rapid adoption of GS is its attractiveness for addressing several components of the breeder's equation simultaneously: Shorten the breeding cycle by replacing phenotypic evaluation steps through a genomic evaluation, increasing accuracy by integrating information from relatives and multiple environments, and increasing selection intensity in case that genotyping is cheaper than phenotyping.

Advances in genome analysis of major crops over the past 15 years led to the availability of a vast number of molecular markers, a pre-requisite for GS application. New genotyping technologies reduced costs of genotyping to a fraction of the costs of phenotyping an individual in field trials.

As a consequence of these developments, GS influenced the design of breeding schemes. With this tool at hand, predictive breeding is used to plan crosses, to reduce breeding cycle length, and to select for more stable performance using multi-year training sets. Genomic prediction is now practiced on many complex traits including yield, quality, biotic, and abiotic stress.

For instance in sugar beet breeding, GS has become an essential component to address the trait “sugar yield,” which is a composite of “sugar content” and “yield.” These two traits are addressed by both (i) within cycle prediction, which allows higher selection intensity and (ii) across cycle prediction, which allows early selection. Predictive ability in each breeding program is constantly monitored. Besides routine application, KWS does very active research to further enhance the efficiency of this tool. Two factors have been the focus of genomic prediction research: chip design and size and composition of training sets. For instance, for sugar beet, we saw that approximately 2,000 markers are sufficient for genomic prediction, potentially due to high linkage disequilibrium in the breeding material. The required training set size is highly dependent of the relationship between training set and prediction set as well as the heritability of the trait. We observe a diminishing return on prediction accuracy for the phenotype of sugar yield when having more than 300 individuals in the training set (which may also be a consequence of the high linkage disequilibrium in breeding populations).

Today, GS has become a routine application in breeding programs at KWS. Thousands of GS analyses are performed every year. Therefore, KWS has optimized genotyping processes and analysis pipelines. With GS being implemented widely in all breeding programs, KWS is extending prediction methods using artificial intelligence and genotype by environment (GxE) interactions.

## Genomic Selection at the International Maize and Wheat Improvement Center (CIMMYT)

CIMMYT has started to explore GS more aggressively as a new breeding tool since 2010 (de los Campos et al., 2009; Crossa et al., 2010, 2019; Dreisigacker et al., 2021). The estimation of GEBVs for the germplasm is routinely implemented for the maize and the wheat program, but it is a decision of the respective breeder which weight is given to this information in the selection process. The initial focus of GS application has been on greater selection intensity in stage I yield trials by predicting the GEBVs of germplasm which had not been included in the trials. Recent projects aim to use GS for early selection and to shorten cycle time. Standardized workflows for data storage, processing, and subsequent analyses are currently advanced by the Excellence in Breeding (EiB) platform and various projects at CIMMYT and other CGIAR centers. CIMMYT has also worked on genomic prediction of traits of germplasm bank accessions (Crossa et al., 2016) to explore its potential for harnessing genetic resources (Martini et al., 2021). The center has built the basis for more informed screening of novel allelic diversity in the germplasm collection by genotyping a substantial part of the available accessions (Sansaloni et al., 2020).

The question which impact GS had on the annual genetic gain for yield across breeding pipelines is more difficult to answer than for the dairy cattle example presented above. Estimates of genetic gain vary and GS has been used to different extend across breeding pipelines. Since programs introduced GS gradually, it is difficult to separate a potential increase in genetic gain due to the use of GS, from other aspects which may have improved the breeding pipelines. A recent publication by Gerard et al. (2020) reports estimated yearly selection gains of 0.93% for low-rainfall environments and 3.8% for high-rainfall environments for the period of 2007–2016 for grain yield in wheat. However, we cannot clearly attribute the credit of this selection gain to GS, since this period is too short after GS has been implemented. However, several dedicated experiments in maize outlined the potential of GS. For instance, Beyene et al. (2015) used GS to select from bi-parental maize populations for yield under drought stress and reported a higher selection gain than for conventional breeding methods. Comparing to previous studies, the authors concluded that “the average gain observed under drought in our study using GS was two- to fourfolds higher than what has been reported from conventional phenotypic selection under drought stress.” Moreover, CIMMYT's Global Maize Program designed a rapid cycle genomic selection (RCGS) of multi-parental crosses (Zhang et al., 2017). Two cycles per year were performed, and the authors found that “the genetic gains from the RCGS […] are at the same or higher level than those observed in other studies under phenotypic selection […].” Also, Beyene et al. (2019) compared selection gain of phenotypic selection (PS) and GS for two different environments (well-watered and water stressed) and observed a higher selection gain for PS for well-watered conditions, and a higher selection gain for GS under water stress. The authors highlighted that GS provides “the potential to bypass stage I trial evaluation and move material directly into stage II” which “would reduce both the costs and cycle time but will require accurate predictions from training sets composed of historical data” (Beyene et al., 2019). This potential to reduce cycle time has not yet been included in the study.

## Implementation of Genomic Selection CGIAR-Wide

The CGIAR has entered a phase of pushing the application of GS for all crops, from maize to bananas (Nyine et al., 2017; Wolfe et al., 2017; Ahmadi et al., 2020; Gemenet et al., 2020; Atanda et al., 2021). The EiB platform provides technical assistance and practical guidelines for the implementation of GS and the modernization of breeding programs (see for instance Covarrubias-Pazaran et al., 2021). Before EiB, several initiatives advanced the use of GS in specific crops. For example, the NextGen Cassava project took important steps toward the successful implementation of GS for root, tuber, and banana (RTB) crops (Wolfe et al., 2017; Maxmen, 2019). Those steps included the development of a robust database system, matching the genotyping logistics with the growing season, and automating analytical pipelines. Similar steps have been taken by initiatives at IRRI and CIMMYT (Crossa et al., 2017; Gao et al., 2020).

Crops currently using GS to reduce cycle time are cassava and maize (Atanda et al., 2021; Esuma et al., 2021). Genomic selection is being used to increase selection intensity in cassava, maize, rice, and wheat (Ahmadi et al., 2020; Dreisigacker et al., 2021). Finally, GS is used for increasing the selection accuracy of yield trials by all the aforementioned and yams (Agre et al., 2018). Other crops, including beans, pulses, forages, bananas, and potato are developing and validating the necessary logistics and tools to manage the data, genotyping, analytical pipelines, and costs. This picture is rapidly changing since the ambition of all breeding programs in the CGIAR is to use genome-assisted prediction methodologies to reduce the length of the breeding cycle to 2–3 years.

## Conclusion

### Dairy Cattle Breeding Compared to Plant Breeding

Genomic selection was adopted in dairy cattle breeding almost instantly after genotyping costs dropped below the anticipated break-even point, presumably because the routine use of pedigree-based predictions, and a culture of centrally processing data of fragmented production units, had already been established (Schaeffer, 1994; Wiggans et al., 2017).

In contrast, plant breeding programs are traditionally dedicated to more specific geographical regions aiming to adapt the germplasm to certain environmental conditions, and the data used for selection decisions have almost exclusively focused on the most recent trials of the respective program. An overarching approach for handling data across programs or selection cycles had not been necessary. Moreover, pedigree information had hardly been used for pedigree-based predictions, since the pedigree information has often been incomplete and “relatively wide” crosses of unrelated material have been used (Dreisigacker et al., 2021). Moreover, a PEBV may not provide additional information, since it cannot capture the segregation within a family generated by a certain cross.

Also, plant breeders traditionally tend to focus on product development that is on identifying varieties, rather than on population improvement, that is identifying parents for new crosses. In other words, breeders are more interested in the genotypic value comprising the complete genetic contribution to the phenotype than in the additive genetic value (the breeding value). A focus on the latter is natural in dairy breeding, where the sire's breeding value is defined indirectly by the performance of its offspring, not by its own phenotype (Mrode, 2014).

Only in recent years some concepts from animal breeding, such as the focus on the breeding value, have been transferred in more formal and more rigorous ways to plant breeding. An example is the separation of population improvement from product development (Gaynor et al., 2017) which allows to focus on the breeding value for the population improvement step. The impact of this paradigm shift on genetic gain is to be observed in coming decade(s).

### Public Compared to Private Plant Breeding

In general, the timelines for the exploration of the potential of GS were relatively similar between the considered public and private plant breeding organizations. CIMMYT and the CGIAR are public research organizations that also pursue the publication of novel, creative approaches, and follow in parts a (research) project-based organization. In contrast, private institutions naturally tend to focus more on the standardization and optimization of routine processes for GS, which may have had a lower priority in the public sector. The EiB platform and associated projects are currently addressing a stronger standardization of data storage and related analysis pipelines. Moreover, the project-based organization in public institutions comes with a variance in funding which leads to challenges for mid to long-term planning on the use of GS.

Finally, CGIAR centers are plant improvement-breeding centers that focus on delivering germplasm to National Agricultural Research institutions (NARs), in particular in Africa and Asia. This implies other priorities for traits, different frameworks for the evaluation of material, and different cost structures compared to, for instance, a commercial program in North America. The economics of implementing GS may therefore differ from those at private companies.

Overall, we think that the advent of GS has provided a tipping point to catalyze the ongoing reform of plant breeding institutions to data processing focused organizations. This transformation will leverage both the historic data resources amassed and the data generated annually to more effectively drive breeding decisions. However, with the increasing number of phenotypic records, and genotypic and environmental information, we now face the challenge of how to use “big data” most efficiently.

## Author Contributions

MG and VW wrote the section about GS at KWS SAAT SE & Co. KGaA. CS, CP, SJH, SD, PP-R, JC, and JM wrote the section on GS at CIMMYT. GC-P wrote the section about CGIAR-wide implementation of GS. Authors from vit (SR and JH) wrote the section about GS in dairy cattle breeding. All authors contributed to the conclusion. MG and JM organized the joint effort.

## Conflict of Interest

MG and VW are employed by the company KWS SAAT SE & Co. KGaA. Moreover, JH and SR are employed by IT Solutions for Animal Production (vit). The remaining authors declare that the research was conducted in the absence of any commercial or financial interest that could be construed as potentially conflicting with the research.

## Publisher's Note

All claims expressed in this article are solely those of the authors and do not necessarily represent those of their affiliated organizations, or those of the publisher, the editors and the reviewers. Any product that may be evaluated in this article, or claim that may be made by its manufacturer, is not guaranteed or endorsed by the publisher.
